# Ulcerative colitis in the postpartum period

**DOI:** 10.4322/acr.2020.187

**Published:** 2020-09-02

**Authors:** Mitsuro Chiba, Tsuyotoshi Tsuji, Masafumi Komatsu, Hiroyuki Watanabe, Masato Takahashi

**Affiliations:** a Akita City Hospital, Division of Gastroenterology. Akita City, Japan.; b Akita Kosei Medical Center, Division of Gastroenterology. Akita city, Japan.; c Akita Kosei Medical Center, Division of Pathology, Akita City, Japan.

**Keywords:** Colitis, ulcerative, postpartum period, diet therapy, labor onset, environment

## Abstract

We describe a scarcely reported case in which ulcerative colitis (UC) occurred in the postpartum period. The aims of this case report are to reinforce the recent assertion that a diet is a ubiquitous environmental factor in inflammatory bowel disease (IBD) and that a plant-based diet (PBD) is recommended for IBD. A 29-year-old woman normally delivered her first child. She first noticed bloody diarrhea 4.5 months after delivery. She was diagnosed with UC (left-sided colitis, moderate severity). Sulfasalazine induced remission. She then experienced and learned about PBD during an educational hospitalization. She resumed breast-feeding and stopped medication. An interview and questionnaire revealed a change in her diet 3 months after delivery, from a sound diet (plant-based diet score: 25) to an unhealthy diet (score: 9). It happened along with a change in residence, from her parent’s home where her mother prepared traditional Japanese meals to her home where she prepared meals by herself. A feeling of release from childbirth prompted her to eat sweets and cheese despite being aware that the quality of the meals deteriorated. We described a scarcely reported case in which UC occurred in the postpartum period. It happened along with a change in her diet, from a sound diet to an unhealthy diet due to a feeling of release from childbirth. She replaced an omnivorous diet by PBD and stopped medication. The critical role of diet is largely ignored by healthcare professionals. We believe that greater appreciation of diet will change and improve management of IBD.

## INTRODUCTION

The incidence of inflammatory bowel disease (IBD), a collective term for ulcerative colitis (UC) and Crohn’s disease (CD), has been increasing over time and expanding to different regions around the world, indicating that IBD is a global disease.^1^ Rapid increase in IBD has been observed in Japan. The incidence and prevalence of UC in 1991 were 1.95/100,000 population and 18.12/100,000 population, respectively.^2^ The prevalence increased to 63.6/100,000 in 2005.^3^ The incidence and prevalence of CD in 1991 were 0.51 and 5.85/100,000 population, respectively.^2^ The prevalence increased to 21.2/100,000 in 2005.^3^ The number of patients receiving public medical aid for UC and CD in Japan were 2,546 and 258, respectively in 1977. These are 167,872 and 42,789, respectively in 2016.^4^ These are 66-fold and 166-fold increases in UC and CD, respectively, during the past 40 years.

Like many other diseases, IBD is a polygenic disease thought to be triggered by environmental factors.^1^ Among various environmental factors underlying IBD, a westernized diet in an affluent society can be identified as a ubiquitous environmental factor.^5^ We have provided plant-based diet (PBD) for IBD inpatients instead of a low residue diet or omnivorous diet since 2003.^6^ PBD incorporates many plant foods such as vegetables, fruits, beans, seeds, and nuts while minimizing animal foods (meat, fish), processed foods, and oils.^7^ There are various types of PBD depending on the degree of animal foods exclusion: vegan, lacto-ovo-vegetarian, semi-vegetarian, and pesco-vegetarian.^7^ Based on our far better outcomes by incorporating PBD for IBD than those in current practices, we for the first time recommended PBD for IBD.^6^ PBDs are listed as variations of USDA healthy eating patterns and are recommended to the public to prevent common chronic diseases.^7^ ^,^ ^8^

Although increased relapse in UC during the postpartum period (6 months) has been reported,^9^ there is scarce knowledge on the onset of UC during the postpartum period. Here we report a case in which UC developed after dietary change from a prudent to an unhealthy diet during the postpartum period. The aims of this case report are to reinforce the recent assertion that a diet is a ubiquitous environmental factor in IBD and that PBD is recommended for IBD.

## CASE REPORT

**Figure 1:**
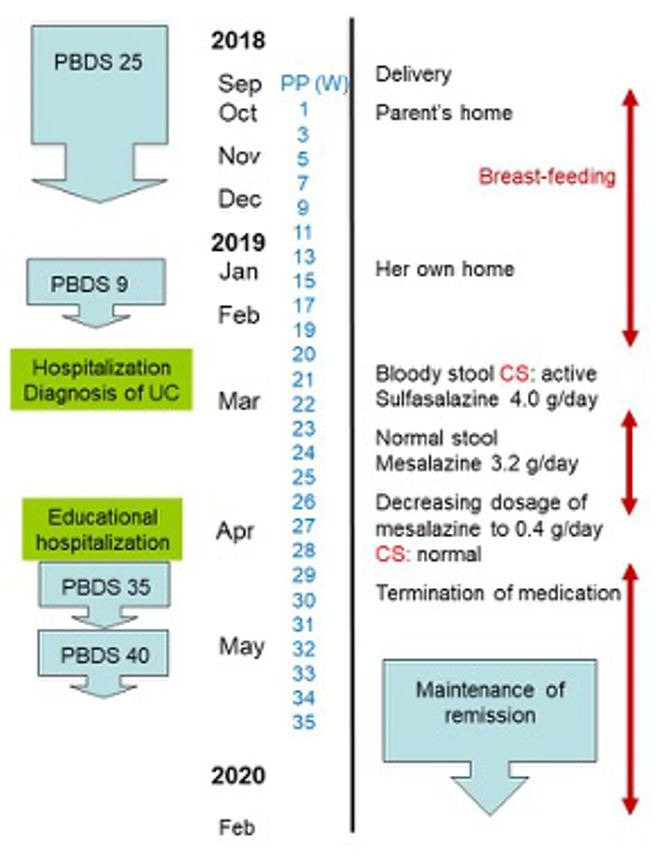
Timeline of case. PBDS = Plant-based diet score; UC = ulcerative colitis; PP = postpartum; W = week; CS = colonoscopy.

A 29-year-old homemaker spontaneously delivered her first child at full term in the end of September 2018 (Figure 1).

She breast-fed the baby. Three months later, she noticed a tendency toward constipation and distress in the epigastrium. She first noticed diarrhea mixed with blood at 20 weeks postpartum (Figure 1). The number of diarrhea episodes increased to 20 times/day in a few days and abdominal pain began to appear. A local doctor referred her to a tertiary care hospital, where she was hospitalized at 21 weeks postpartum (Figure 1).

Her past history was noncontributory except for palm exanthema at the age of 26. There was no family history of IBD. She reported not eating any foods that would likely cause diarrhea. Her family (husband and baby) did not have diarrhea. She did not take any supplements or medications.

**Figure 2:**
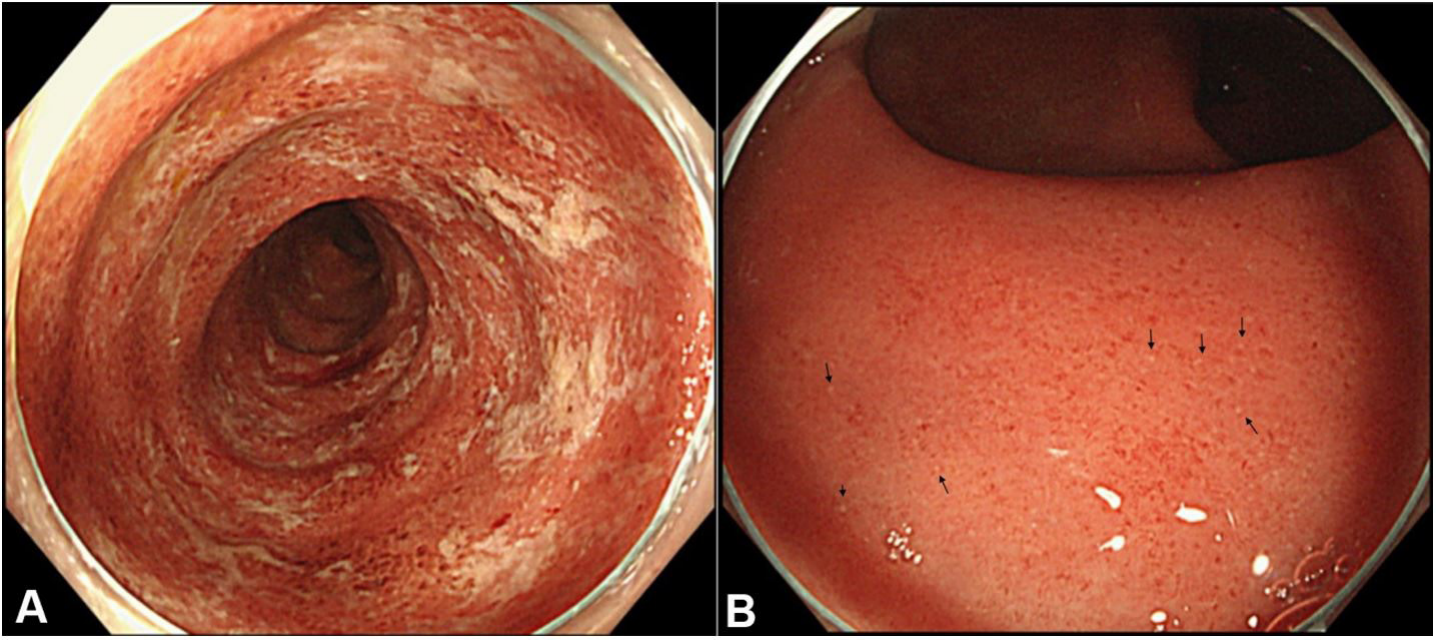
Photograph of colonoscopy at 21 weeks postpartum. **A –** Diffuse erythematous mucosa with patchy mucous adherences was observed in the sigmoid colon; **B –** Diffuse inflammation without normal vascular pattern was observed in the rectum. Multiple yellow-white color spots (erosions) (arrows) were observed.

**Figure 3:**
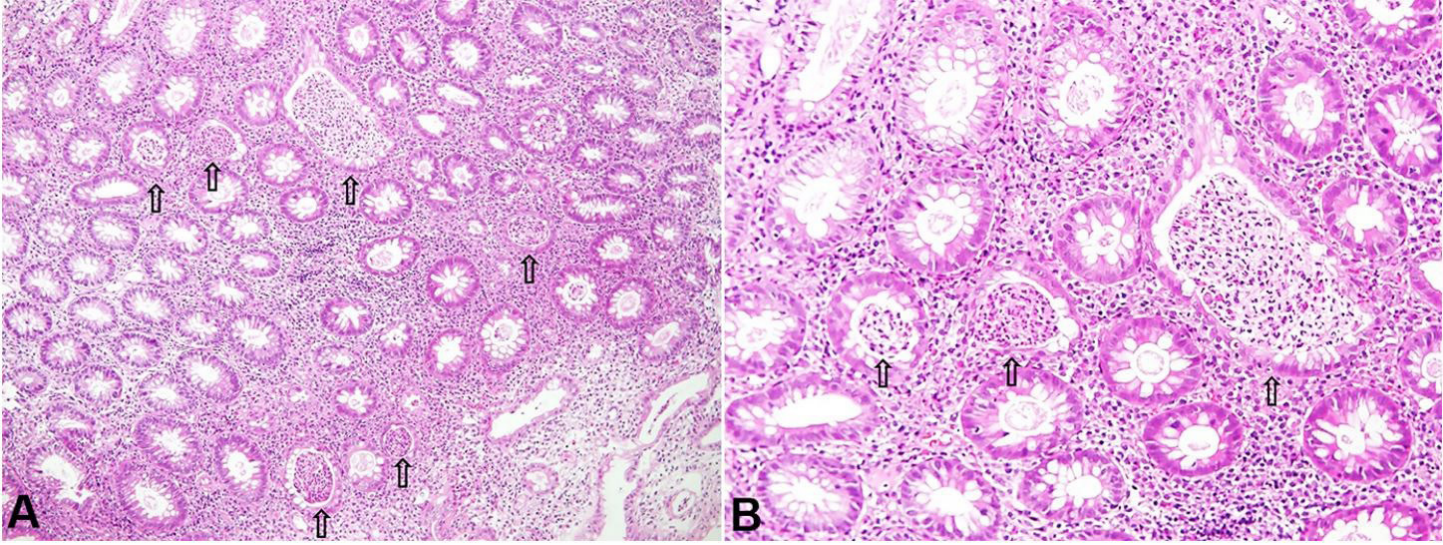
Photomicrographs of the biopsy specimen from the sigmoid colon. Crypt abscesses (arrows), goblet cell depletion, and mononuclear cell infiltration were observed. (**A**) (H&E, 100X); (**B**) (H&E, 200X).

Her height was 165 cm and body weight was 60 kg. She was afebrile. Physical examination was noncontributory except for mild tenderness in the left lower abdomen. Neither anal fistula nor anal skin tag was observed. Blood test disclosed a mild elevation of C-reactive protein (Table 1). Colonoscopy revealed diffuse inflammation from the rectum to the proximal descending colon with multiple yellow-white color spots^10^ (Figure 2). Ulcer was not observed. Biopsy specimens revealed crypt abscesses, goblet cell depletion, and mononuclear cell infiltration (Figure 3), which were consistent with findings of UC. Stool culture for pathogen, CD Chek (Techlab C. Diff Quik Chek Complete, Techlab Inc, VA, USA), and cytomegalovirus antigenemia^11^ were negative. At this point, moderate UC (initial episode) with left-sided colitis in the extent^12^ was diagnosed. Sulfasalazine 4.0 g/day was prescribed (Figure 1). Her symptoms gradually improved, then bloody stool disappeared. She was discharged after 2 weeks hospitalization. Soon after discharge, sulfasalazine was replaced by mesalazine (3.2 g/day),^13^ and she resumed breast-feeding (Figure 1). Because she wanted to be free from medication, she asked her doctor to refer her to another tertiary hospital based on the information of PBD in IBD.

**Table 1:** Laboratory data

Item	(Normal range)		Unit		21 wks PPa		22 wks PP		23 wks PP		27 wks PPb	35 wks PP
Hemoglobin	11.6-14.8		g/dl		11.7		10.6		10.9		11.5	12.5
White blood cell	3300-8600		/mm^3^		7800		6300		5500		4500	4250
Neutrophil	38-72		%		61.3							
Lymphocyte	22-54		%		19.7							
Monocyte	2-7		%		11							
Eosinophil	0-5		%		7.4							
Basophil	0-2		%		0.6							
Platelet	15.8-34.8		x 10^4^/mm^3^		25.7		29.5		30.2		18.1	21.5
Total protein	6.6-8.1		g/dl		7.5						7.8	7.6
Albumin	4.1-5.1		g/dl								4.7	4.5
Sodium	138-145		mEq/l		141							
Potassium	3.6-4.8		mEq/l		3.9							
Chloride	101-108		mEq/l		103							
Choline esterase	201-421		IU/mL				192		244		309	319
C-reactive protein	0-0.14		mg/dl		0.44		0.23		0.02		<0.02	<0.02
Erythrocyte sedimentation rate	≤15		mm/hr						22		18	11
Fecal occult blood	<100		ng/mL								<50	

^a^Admission for active ulcerative colitis;

^b^Educational hospitalization for ulcerative colitis. Blueprint shows abnormal value. Blanks mean that the item was not tested. PP = postpartum.

She underwent 12 days of educational hospitalization for UC^14^ at 27 weeks postpartum (Figure 1). At that time, there were no symptoms. C-reactive protein was normal and fecal occult blood was negative (Table 1). A lacto-ovo-semi-vegetarian diet (1700 kcal/day) with fish once a week and meat every other week,^15^ a kind of PBD, was provided. She experienced PBD and had a dietary guidance of PBD. Colonoscopy at the end of hospitalization revealed restoration of vascular patterns which confirmed endoscopic remission of UC (Figure 1). She requested a lower dose of medication. Considering her excellent condition and our assertion that diet is generally more important than medication in the quiescent stage in IBD,^15^ the mesalazine dose was gradually decreased to 0.4 g/day on discharge (Figure 1). She was advised to continue PBD after discharge.^14^

An interview and questionnaire on lifestyle and dietary habits^16^ revealed a dietary change during the postpartum period. Midwives recommended Washoku^17^ for pregnancy and for lactation. Washoku is a traditional Japanese diet prevalent before dietary westernization and is similar to a presco-vegetarian diet. After delivery, she stayed at her parent’s home for about 3 months. Her mother prepared Washoku at the midwife's recommendation. She subsequently returned to her own home at 14 weeks postpartum (Figure 1). She had a feeling of being released from childbirth. She prepared meals by herself. Although she was aware that the quality of the meals deteriorated, she had urges to eat sweets and cheese. She felt a tendency of constipation and distress in the epigastrium. This dietary change was assessed by plant-based diet score (PBDS).^16^ The score dropped from 25 to 9 (Table 2, Figure 1).

**Table 2:** Plant-based diet Score (PBDS) for Japanese patient with inflammatory bowel disease

		Scoring by frequency of								Present case			
Food group		serving days/week								Baseline	After	Educational	One month after
		Dai ly		3-5		1-2		Rarely		(until 3 months	3 months	hospitaliza tion	educational
										postpartum)	Postpar tum	(12 days)	hospitaliza tion
Positive score													
Vegetables		5		3		1		0		5	3	5	5
Fruits		5		3		1		0		5	3	5	5
Pulses		5		3		1		0		5	5	5	5
Potatoes/starches		5		3		1		0		1	1	5	5
Rice		5		3		1		0		5	5	5	5
Miso soup		5		3		1		0		5	1	5	5
Green tea		5		3		1		0		0	0	0a	5
Yoghurt (plain)		5		3		1		0		5	5	5	5
**Negative score**													
Meat		-5		-3		-1		0		-1	-1	0	0
Minced or processed meat		-5		-3		-1		0		-1	-1	0	0
Cheese/butter/margarine		-5		-3		-1		0		-1	-5	0	0
Sweets/ice cream/milk shake		-5		-3		-1		0		-1	-5	0	0
Soft drinks (cola/carbonated beverages/juice)		-5		-3		-1		0		0	-1	0	0
Alcohol		-5		-3		-1		0		0	0	0	0
Bread		-5		-3		-1		0		0	-1	0	0
Fish		-2		-1		0		0		-2	0	0	0
**Plant-based diet score (PBDS)**										**25**	**9**	**35**	**40**

^a^Green tea is recommended to drink at home but is not provided at a hospital.

After the educational hospitalization, she was glad to be able to resume breast-feeding. She was well, then she completely stopped taking her medication 3 weeks after discharge. Her PBDS one month after the discharge (35 weeks postpartum) was 40, the highest score (Table 2). The values of hemoglobin and erythrocyte sedimentation rate became normal (Table 1). She has remained in remission without medication to the present (February 2020).

The patient has provided informed consent for publication of the case.

## DISCUSSION

Although the etiology of IBD is generally stated as unknown, we first regarded IBD as lifestyle disease mediated mainly by westernized diet.^15^ PBD was designed to combat dietary westernization.^6^ ^,^ ^15^ It is apparent now that diets shape gut microbiota.^18^ Recently, basic research on the gut microbiome has provided a rationale for how PBD is superior to westernized diets.^19^ ^-^ ^21^ Westernized diet (high in fat, animal protein and sugar, low in dietary fiber) tend to decrease microbial diversity (dysbiosis), while PBD (low in fat, animal protein and sugar, high in dietary fiber) tend to increase microbial diversity.^19^ ^-^ ^21^ This difference in microbiota results in differences in microbial metabolites. Westernized diets result in increased production of ammonia, indols, phenols, and sulphide that may be detrimental to our health. In addition, they result in decreased production of short-chain fatty acids like butyrate. Short-chain fatty acids, particularly butyrate, have diverse beneficial effects in nutrition, immunity, and epithelial barrier function (enhanced mucous secretion and increased antimicrobial peptide). PBD result in increased production of short-chain fatty acids. Altogether, westernized diet are pro-inflammatory while PBD is anti-inflammatory.^19^ ^-^ ^21^ These observations indicate that westernized diets are susceptibility to not only IBD but also other chronic diseases.

Focusing on diets, we reported cases with new onset of IBD during a change in dietary habits: UC during a change of lifestyle,^22^ during low-carbohydrate weight-loss diet,^23^ in the second trimester after emesis gravidarum,^24^ and CD after moving to Tokyo.^25^ The present case of UC in the postpartum period is another example demonstrating that diet is a critical environmental factor in IBD.^5^ PBDS evaluate adherence to PBD: a higher PBDS indicates greater adherence to PBD.^16^ The present case’s PBDS after 3 months postpartum was 9 (Table 2, Figure 1), which was comparable to the mean baseline PBDS (10.9) in 158 UC patients.^16^

When PBD was first provided to IBD patients, we assumed that diet was generally more important than medication to prevent relapse in the quiescent phase,^15^ and our findings have shown that to be true. Relapse rates in both UC and CD were far lower than those with current medication.^6^ ^,^ ^14^ ^,^ ^15^ ^,^ ^26^ PBDS in these patients were significantly higher than baseline PBDS not only in the short-terms of less than 2 years after discharge but also even in the long-term: median 3.9 years^14^ and 6.4 years^26^. This certifies that they consumed more of the recommended food and consumed less of the food that was discouraged compared with baseline. Although current practice recommends adherence to medication,^27^ we emphasize that diet is more important than medication in the quiescent phase of IBD. Therefore, medication is withdrawn whenever patients have confidence in relapse prevention. The maximum PBDS score, 40 in the present case one month after educational hospitalization (Table 2, Figure 1), seems to be a reflection of her strong conviction that the disease is suppressed by sound lifestyle.

Unfortunately, lifestyle medicine including dietary habits is not fully appreciated in current medicine.^28^ ^,^ ^29^ Appreciation of lifestyle medicine is needed to prevent and treat current common chronic diseases in affluent society.

In conclusion, we described a scarcely reported case in which UC occurred in the postpartum period. It happened along with a change in her diet, from a sound diet to an unhealthy diet due to a feeling of release from childbirth. This case supports the recent assertion that a diet is a ubiquitous environmental factor in IBD^5^ and that PBD is recommended for IBD^6^. This observation should encourage researchers to explore the PBD properties in controlled studies.

## References

[1] Kaplan GG, Ng SC (2017). Understanding and preventing the global increase of inflammatory bowel disease. Gastroenterology.

[2] Morita N, Toki S, Hirohashi T (1995). Incidence and prevalence of inflammatory bowel disease in Japan: nationwide epidemiological survey during the year 1991. J Gastroenterol.

[3] Asakura K, Nishiwaki Y, Inoue N, Hibi T, Watanabe M, Takebayashi T (2009). Prevalence of ulcerative colitis and Crohn’s disease in Japan. J Gastroenterol.

[4] Japan (2019). Japan.

[5] Chiba M, Nakane K, Komatsu M (2019). Westernized diet is the most ubiquitous environmental factor in inflammatory bowel disease. Perm J.

[6] Chiba M, Ishii H, Komatsu M (2019). Recommendation of plant-based diet for inflammatory bowel disease. Transl Pediatr.

[7] Tuso PJ, Ismail MH, Ha BP, Bartolotto C (2013). Nutritional update for physicians: plant-based diets. Perm J.

[8] U.S. Department of Health and Human Services (2015). The 2015 dietary guidelines for Americans.

[9] Pedersen N, Bortoli A, Duricova D (2013). The course of inflammatory bowel disease during pregnancy and postpartum: a prospective European ECCO-EpiCom study of 209 pregnant women. Aliment Pharmacol Ther.

[10] Travis SPL, Schnell D, Krzeski P (2013). Reliability and initial validation of ulcerative colitis endoscopic index of severity. Gastroenterology.

[11] Chiba M, Abe T, Tsuda S, Ono I (2013). Cytomegalovirus infection associated with onset of ulcerative colitis. BMC Res Notes.

[12] Satsangi J, Silverberg MS, Vermeire S, Colombel J-F (2006). The Montreal classification of inflammatory bowel disease: controversies, consensus, and complications. Gut.

[13] Mahadevan U, Robinson C, Bernasko N (2019). Inflammatory bowel disease in pregnancy clinical care pathway: a report from the American Gastroenterological Association IBD Parenthood Project Working Group. Gastroenterology.

[14] Chiba M, Nakane K, Tsuji T (2018). Relapse prevention in ulcerative colitis by plant-based diet through educational hospitalization: a single-group trial. Perm J.

[15] Chiba M, Abe T, Tsuda H (2010). Lifestyle-related disease in Crohn’s disease: relapse prevention by a semi-vegetarian diet. World J Gastroenterol.

[16] Chiba M, Nakane K, Takayama Y (2016). Development and application of a plant-based diet scoring system for Japanese patients with inflammatory bowel disease. Perm J.

[17] Government of Japan (2019). Washoku: traditional dietary cultures of the Japanese.

[18] De Filippo C, Cavalieri D, Di Paola M (2010). Impact of diet in shaping gut microbiota revealed by a comparative study in children from Europe and rural Africa. Proc Natl Acad Sci USA.

[19] David LA, Maurice CF, Carmody RN (2014). Diet rapidly and reproducibly alters the human gut microbiome. Nature.

[20] Sonnenburg ED, Sonnenburg JL (2014). Starving our microbial self: the deleterious consequences of a diet deficient in microbiota-accessible carbohydrates. Cell Metab.

[21] Simpson HL, Campbell BJ (2015). Review article: dietary fiber-microbiota interactions. Aliment Pharmacol Ther.

[22] Chiba M, Yamashita T, Kon H (2005). A change in lifestyle was thought to result in onset of ulcerative colitis: case report. Akita Med J..

[23] Chiba M, Tsuda S, Komatsu M, Tozawa H, Takayama Y (2016). Onset of ulcerative colitis during low-carbohydrate weight-loss diet and its treatment with plant-based diet: a case report. Perm J.

[24] Chiba M, Sugawara T, Komatsu M, Tozawa H (2018). Onset of ulcerative colitis in the second trimester after emesis gravidarum: treatment with plant-based diet. Inflamm Bowel Dis.

[25] Chiba M, Sugawara T, Morikawa Y (2006). Onset of Crohn’s disease after moving to Tokyo: maintenance of remission by semi-vegetarian diet: a case report. Digestion & Absorption.

[26] Chiba M, Nakane K, Tsuji T (2019). Relapse prevention by incorporative plant-based diet in induction phase for ulcerative colitis: A single group trial. Perm J.

[27] Kane SV (2007). Overcoming adherence issues in ulcerative colitis. Gastroenterol Hepatol.

[28] Bodai BI, Nakata TE, Wong WT (2018). Lifestyle medicine: a brief review of its dramatic impact on health and survival. Perm J.

[29] Chiba M, Nakane K, Komatsu M (2018). Lifestyle medicine in inflammatory bowel disease. Perm J.

